# Complicated Diagnosis and Treatment of Rare Painless *Acanthamoeba* Keratitis

**DOI:** 10.3390/jcm14134763

**Published:** 2025-07-05

**Authors:** Dominika Wróbel-Dudzińska, Marta Ziaja-Sołtys, Beata Rymgayłło-Jankowska, Monika Derda, Robert Klepacz, Daniel Zalewski, Tomasz Żarnowski, Anna Bogucka-Kocka

**Affiliations:** 1 Department of Diagnostic and Microsurgery of Glaucoma, Medical University of Lublin, Chmielna 1 Street, 20-079 Lublin, Poland; 2 Department of Biology with Genetics, Medical University of Lublin, Witolda Chodźki 4A Street, 20-093 Lublin, Poland; 3 Department of Biology and Medical Parasitology, Poznan University of Medical Sciences, H. Swiecicki Street 4, 60-781 Poznan, Poland; 4 Department of Clinical Pathology, Medical University of Lublin, Jaczewskiego 8B Street, 20-090 Lublin, Poland

**Keywords:** *Acanthamoeba* keratitis, *Herpes simplex*, painless, diagnosis, therapy

## Abstract

**Objectives**: The aim was to present the complicated diagnostic and therapeutic process of atypical, painless keratitis caused by a cosmopolitan protozoan of the genus *Acanthamoeba*. **Methods**: This Case Report describes a medical case involving a 48-year-old woman who occasionally wears soft contact lenses and was referred to our hospital for treatment due to deteriorating visual acuity in her left eye. The diagnostic process included the isolation of amoebae from corneal scrapings and the morphological and molecular identification of the etiological agent of the infection. **Results**: After examination, painless atypical keratitis was diagnosed, initially considered recurrent herpetic keratitis. However, antiviral treatment did not bring about any improvement. Further observation revealed a dense, central, annular infiltrate on the periphery of the cornea. Despite treatment, the corneal infiltrate did not improve and the patient required therapeutic penetrating keratoplasty. Ultimately, the patient underwent combined surgery: corneal transplantation with cataract phacoemulsification and intraocular lens implantation. The postoperative course was uneventful. **Conclusions**: *Acanthamoeba* keratitis should be included in the differential diagnosis of keratitis, even in the absence of its characteristic feature of severe ocular pain, especially in contact lens wearers and patients who have had herpetic keratitis. Infection of the cornea with the *Herpes simplex* type 1 virus causes nerve degeneration, which probably translates into a painless course of *Acanthamoeba castellanii* infection.

## 1. Introduction

*Acanthamoeba* keratitis (AK) is a severe corneal infection caused by pathogenic genotypes of the *Acanthamoeba* genus, which, according to the current scientific literature, often leads to blindness [1,2,3,4]. *Acanthamoeba* sp. is a protozoan from the group of free-living amoeba together with other species, such as *Balamuthia*, *Naegleria*, and *Sappinia* sp. All of them live in soil and lakes, but can also occur in swimming pools and drinking water reservoirs [5]. *Acanthamoeba* sp. exists in two different stages, both of which are infective: the trophozoite, which is able to feed and divide, and the cyst, which is a dormant stage resistant to harsh conditions [5,6,7]. This free-living amoeba is pathogenic, especially to immunocompromised patients [5]. *Acanthamoeba* amoebas have also been detected in contaminated contact lens cleaning solutions and have been confirmed to grow on lenses cleaned with tap water [8].

The genus *Acanthamoeba* was classified according to the 18S rRNA (rDNA) sequence (types T1-T22). *Acanthamoeba castellanii* type T4 was found to be the etiological factor in 94.3% cases of *Acanthamoeba* keratitis. However, genotypes T2, T3, T5, T6, T11, T13, and T15 have also been related to AK and amoebic encephalitis [9,10,11,12,13]. The other listed species, i.e., *A. polyphaga*, *A. culbertsoni*, *A. hatchetti*, *A. rhysodes*, *A. griffini*, *A. quina*, and *A. lugdunensis*, are detected less frequently [14]. In recent years, an increase in the number of infections with this parasite has been observed around the world [15]. The annual incidence of AK worldwide is 2.9 cases per million people per year, but there are significant differences between countries [16]. This represents 2% of all corneal inflammations and is usually one-sided, although it can affect both eyes in lens-wearing patients [9,10].

AK is an infection that poses a major challenge for physicians to diagnose and treat [2]. Clinical manifestations are nonspecific and include tearing, unilateral photophobia, and redness [17,18]. At the beginning of the disease, dendritic epitheliopathy resembling *Herpes simplex* keratitis (HSK) often develops, with or without stroma and endotheliitis [19]. Therefore, the initially nonspecific acute presentation of AK is often misdiagnosed as adenoviral herpetic, bacterial, mycotic keratitis, or corneal epithelial erosion [16,20,21].

The hallmark symptom of *Acanthamoeba* keratitis is exquisite pain, disproportionate to the clinical picture [22]. This pronounced pain is attributed to the exceptional sensitivity of the cornea due to its dense sensory, sympathetic and parasympathetic nerve fiber network, which makes it the best innervated structure in the human body. Most of the sensory innervation of the cornea consists of long ciliary and nasociliary neurons originating from the ophthalmic division of the trigeminal ganglion. These fibers do not contain myelin, making the tissue transparent [23,24,25,26].

It is believed that any disruption to the proper state of corneal innervation, as a result of infection or surgery, can lead to transient or chronic neurotrophic keratopathy (NK) [24,27]. The disease begins spontaneously with the breakdown of epithelial cells, and the impaired healing mechanisms of the corneal surface increase susceptibility to soreness. A similar pathophysiological process may underlie the pain associated with AK. However, some studies have documented cases of ocular acanthamoebiasis presenting without pain [28,29,30]. Decreased corneal sensation has previously been reported in the context of ocular infection with the *Herpes simplex* type 1 virus and as a consequence of iatrogenic injury during neurosurgical procedures [26,31]. The underlying mechanisms of this phenomenon may also play a role in the atypical, painless clinical presentation of AK; nevertheless, the underlying cause of the absence of pain in these cases remains inconclusive.

Our aim is to present the diagnostic and therapeutic process of painless keratitis caused by *Acanthamoeba* and to summarize the current literature data on this type of ocular infection.

## 2. Detailed Case Description

**Figure 1:**
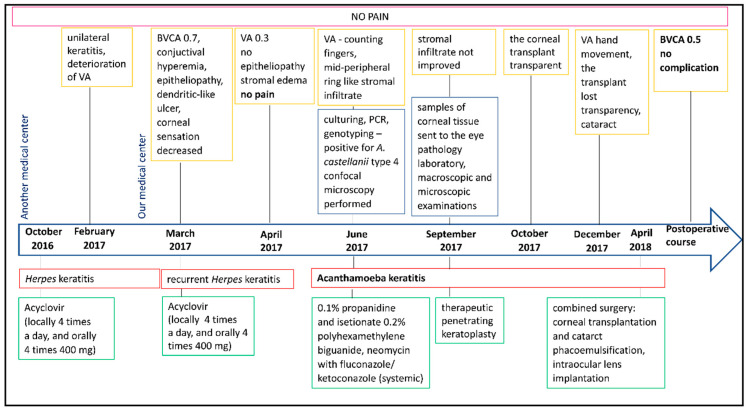
Timeline of the diagnostic procedures and treatment decisions in the patient’s case.

**Figure 2:**
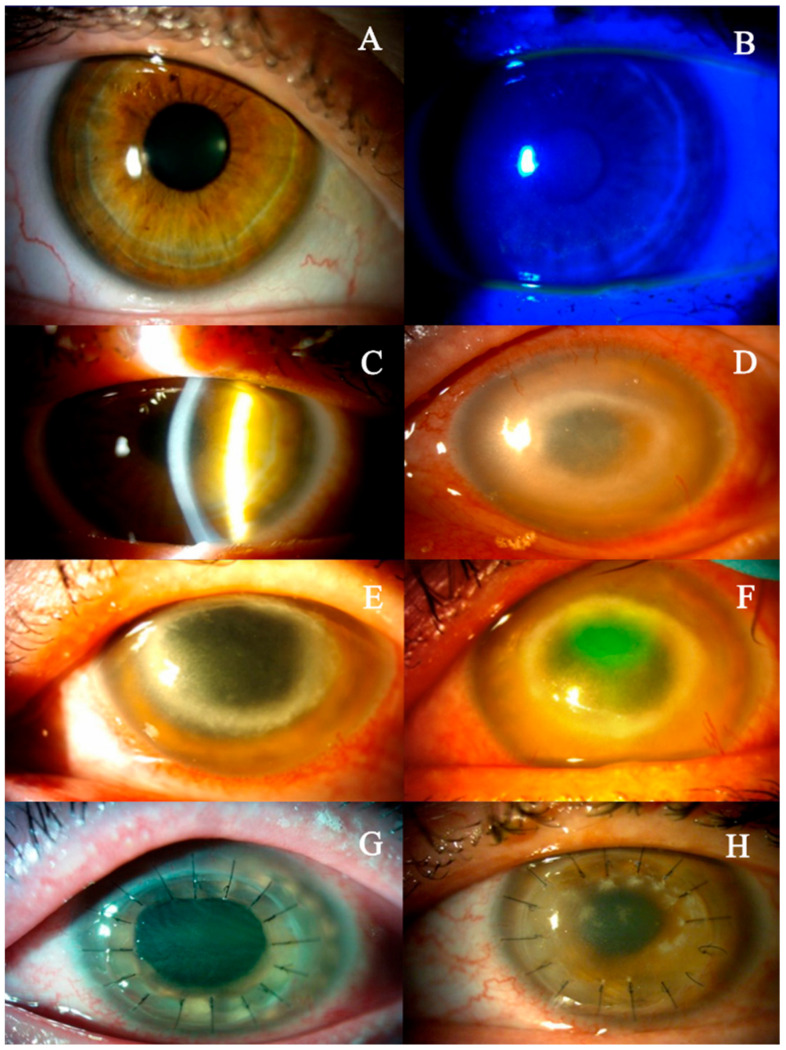
(**A**) Conjunctival hyperemia. (**B**) Subtle epithelial irregularities resembling a dendritic-like ulcer located in the lower corneal hemisphere. (**C**) Stromal edema. (**D**) Dense mid-peripheral ring-like stromal infiltrate. (**E**) Ring infiltration. (**F**) Extensive stromal infiltrate and epithelial defect. (**G**) Clear corneal transplant. (**H**) Graft lost transparency and developed cataracts.

In March 2017, a 48-year-old female patient with myopia presented to our hospital with unilateral keratitis and a two-week history of decreased visual acuity in the left eye (Figure 1). Her overall health was good. According to the medical history, she occasionally wore contact lenses and had been treated with acyclovir (locally four times a day, and orally four times a day at 400 mg) five months earlier due to herpetic keratitis of the left eye in another medical center. Upon the examination, the best corrected visual acuity (BCVA) was 0.7 in the left eye and 1.0 in the right eye, and the intraocular pressure (IOP) in the left eye was 14 mmHg, in the right eye 13 mmHg. Ocular examination of the left eye revealed mild conjunctival hyperemia and subtle epithelial changes resembling a dendritic-like ulcer located in the inferior corneal hemisphere (Figure 2A,B). The corneal sensation in the left eye was decreased. Recurrent herpetic keratitis was diagnosed and local (four times a day) and oral (four times at 400 mg) acyclovir treatment was initiated. After three weeks (April 2017), despite the slight improvement, that is, no epitheliopathy, stromal swelling was observed in the lower corneal hemisphere. Furthermore, visual acuity in the left eye had declined to 0.3 (Figure 2C). In May 2017, a deeper corneal ulcer was detected in the superior corneal hemisphere. Upon inquiry, the patient reported no ocular pain. There was no abnormal finding in the left eye. During further observations (June 2017), a dense mid-peripheral ring-like stromal infiltrate developed (Figure 2D).

The visual acuity of the affected eye deteriorated to the counting fingers stage. Deep corneal scrapings were collected and labeled as AC55. The scraping sample was inoculated into a non-nutrient agar (BD Difco Ltd., Detroit, MI, USA), poured on a Petri dish and covered with a suspension of the bacterium *Enterobacter aerogenes*. The amoebae were grown in axenic liquid cultures containing 2% Bacto Casiton (Gibco, Life Technologies, Carlsbad, CA, USA) and 10% normal horse serum according to the procedure described by Červa [32]. The plates were incubated at a temperature of 28 °C. After 1–5 days, an increase in the number of amoebae was observed and examined with an inverted microscope at ×200. The amoebae isolated from the cornea were identified according to morphological criteria, measurements of the size of cysts and tests for flagellation [33]. The culture was positive for *Acanthamoeba* sp.

**Figure 3:**
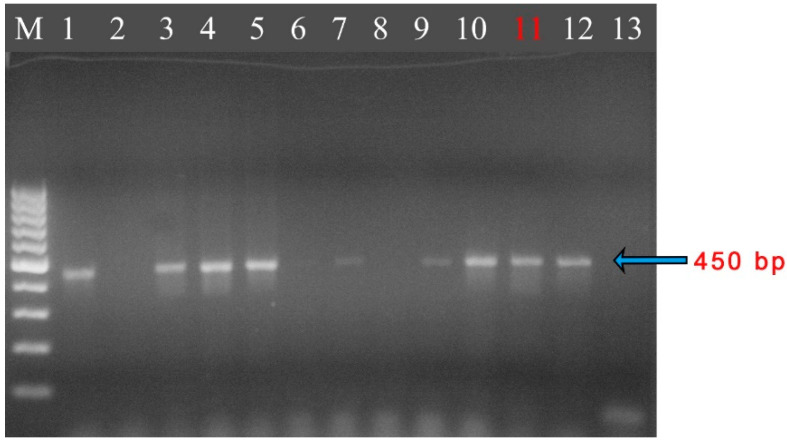
Gel showing the separation of DNA obtained in a PCR using primers JDP1 and JDP2 (450 bp product). Samples: M—standard, 1—control (*A. castellanii*), 2—negative control, 3—*A. castellanii*, 4—*A. rhysodes*, 5—*A. polyphaga*, 6—*Hartmannella vermiformis*, 7—strain IC1, 8–9—*Naegleria* sp., 10—sample AC54, 11—sample AC55, 12—sample AC60, 13—sample AC66. Obtained results confirmed the presence of *Acanthamoeba* sp. in sample AC55. Samples AC54, AC60, and AC66 belong to an independent study.

The polymerase chain reaction (PCR) for *Acanthamoeba* was performed using genus-specific primers previously described by Schroeder et al. [34] (see Supplementary Material for details). DNA was isolated from the corneal scrapings (AC55 sample), and amplicons of the 18S rRNA gene fragment were obtained. The results of the PCR confirmed that the sequences obtained from the AC55 isolate were identical to the *Acanthamoeba* sequences deposited in GenBank (Figure 3). The PCR products were subsequently submitted for sequencing analysis (see Supplementary Material for details). The analysis revealed that the sequences were 100% identical to the parasite’s 18S rRNA gene isolated from the liver of an infected pheasant *Tragopan temminckii* (GQ889265), corneal scrapings (KF318460, DQ087297), a contact lens (DQ087296), and an environmental sample (EU377583) (Table 1) [35]. The *Acanthamoeba* sequences from the isolates obtained from corneal scrapings (AC55) were deposited in GenBank (NCBI) under accession number KP120880. The isolated amoeba strain belonged to genotype T4 of the genus *Acanthamoeba*.

Intensive treatment was initiated, consisting of topical 0.1% propamidine isethionate, 0.2% polyhexamethylene biguanide and neomycin, in combination with systemic antifungal therapy using fluconazole and/or ketoconazole. In July 2017, a large epithelial defect was identified at the border of the stromal ring. After three months of amoebicidal therapy, there was no significant improvement in the stromal infiltrate, necessitating therapeutic penetrating keratoplasty (September 2017) (Figure 2E,F). The patient did not report any pain at any time before the surgery.

**Figure 4:**
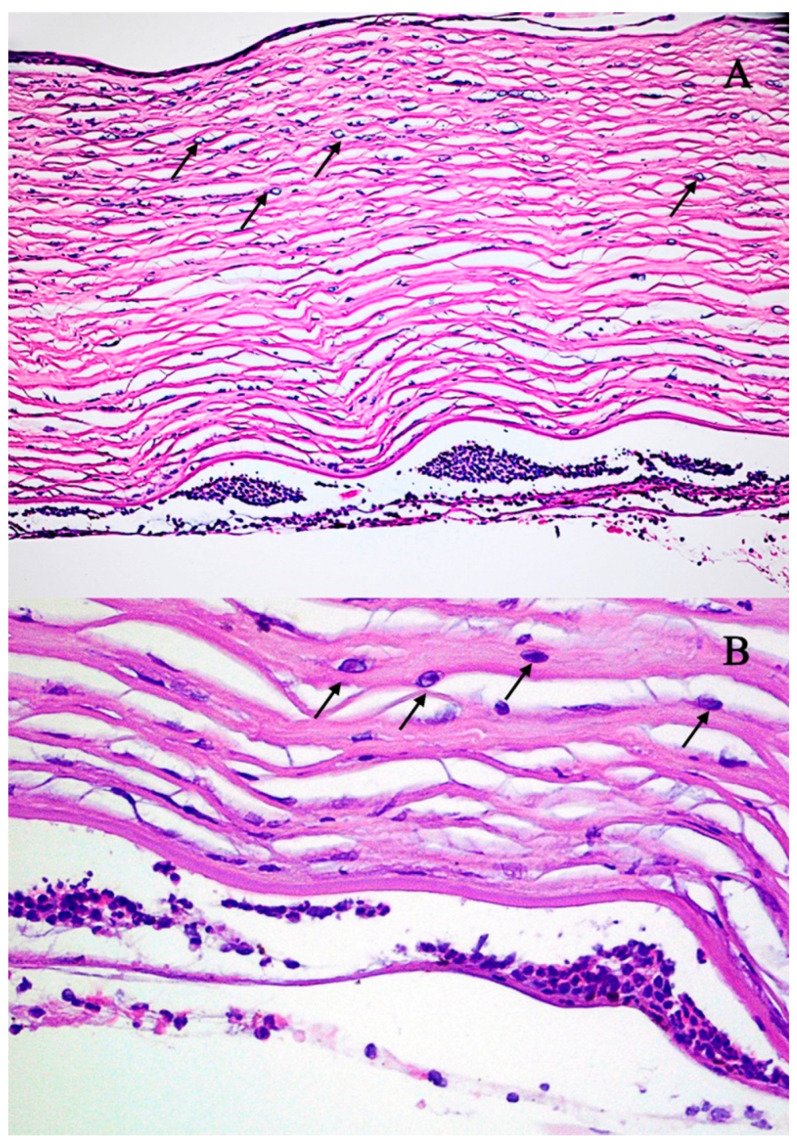
Ocular pathology examination of the patient’s cornea, demonstrating multiple intrastromal *Acanthamoeba* cysts ((**A**), arrows) and trophozoites ((**B**), arrows). Hematoxylin and eosin staining: panel (**A**)—magnification 40×; panel (**B**)—magnification 400×.

**Figure 5:**
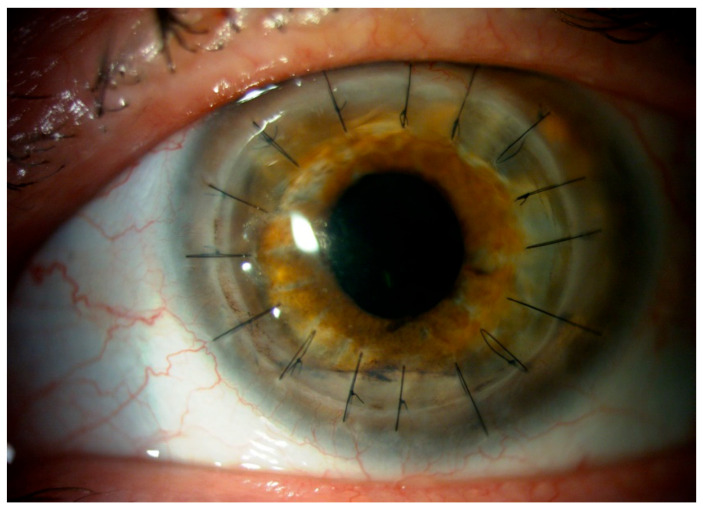
Clear corneal transplant with IOL (intraocular lens) following cataract phacoemulsification.

The tissue was transferred to the eye pathology laboratory. Macroscopic histological examination of the corneal tissue revealed that the specimen consisted of an opaque corneal nodule measuring 8 mm in diameter. Microscopic examination revealed marked attenuation and focal detachment of the corneal epithelium. The stroma exhibited pronounced edema accompanied by focal neutrophilic infiltration. Numerous *Acanthamoeba* cysts and trophozoites were widely distributed throughout the stromal tissue. The descemet membrane appeared structurally intact but was extensively covered with inflammatory cells, predominantly neutrophils. Only a sparse population of corneal endothelial cells was identified (Figure 4A,B). At the initial follow-up visit in October 2017, the ocular examination revealed a transparent corneal transplant secured with 16 Nylon 10/0 sutures (Figure 2G). After three months (December 2017), the transplant lost transparency, and a few sutures were loosened (Figure 2H). Moreover, a cataract was diagnosed in the left eye. At the final follow-up, our patient’s visual acuity was at the hand movement stage. Three months later (April 2018), the patient underwent combined surgery: corneal transplantation along with cataract phacoemulsification and intraocular lens implantation. The postoperative course was without complications, and on the last follow-up visit the best corrected visual acuity was 0.5 (Figure 5).

## 3. Discussion and Review

*Acanthamoeba* keratitis is a potentially vision-threatening corneal infection. Diagnostic challenges primarily stem from the nonspecific clinical signs and symptoms observed during the initial stages of the disease; moreover, this pathology may mimic other infections [17]. The German Acanthamoeba Keratitis Registry has revealed that *Acanthamoeba* keratitis has been misdiagnosed as herpetic (in 47.6% of cases), mycotic (in 25.2%), and bacterial (in 3.9%) keratitis in patients [41]. It is noteworthy that a mixed infection with viruses, bacteria, or fungi is present in approximately 23% of keratitis cases [42]. According to other sources, *Acanthamoeba* infections are polymicrobial, with 12.5% of co-infections with bacteria, 40% of co-infections with fungi, and 5% of triple infections. In addition, co-infections account for 55% of all *Acanthamoeba* infections [43].

So far, 54 cases of painless AK have been reported in the literature. Some studies have shown the tendency of *Acanthamoeba* to co-exist with other microorganisms, which increases their pathogenicity and survival, thus making their treatment more difficult. In total, 16/54 (29.6%) patients wore contact lenses. Infection only with *Acanthamoeba castellanii* was diagnosed in 38/54 (70.37%) patients without concomitant bacterial, viral, or fungal infection. Bacterial co-infection was detected in 13 (81.25%) of 16 patients, and co-infection was caused solely by viruses in 4 of 16 (25%) patients. Co-infection with bacteria and *Herpes simplex* virus was confirmed in one patient. These data are shown in Table 2.

In the literature, the pain in *Acanthamoeba* keratitis is described as severe and disproportionate to the clinical findings during the slit lamp examination [44,45]. Other initial symptoms are often nonspecific [29], which means that *Acanthamoeba* keratitis may often be misdiagnosed as herpetic keratitis or epithelial erosions early in the course of the disease, resulting from the presence of an epithelial defect. Therefore, high clinical suspicion should be maintained for *Acanthamoeba* keratitis in patients presenting with relevant risk factors, even in the absence of ocular pain [46,47].

This case illustrates an atypical presentation of Acanthamoeba keratitis, characterized by the complete absence of ocular pain throughout the disease course. The patient experienced only mild ocular discomfort without any reported pain. The mechanism of painless *Acanthamoeba* keratitis is not completely understood, but it is suggested to be the result of perineuritis, preexisting neurotrophic cornea (such as *Herpes* keratitis), or pretreatment with topical steroids, which mask the clinical signs of this keratitis [30,48,49,50].

**Table 2:** Cases of painless AK reported in the literature; ”+”—yes, “−“—no.

Reference	Number of Cases	Contact Lens Use	*Acanthamoeba castellanii* Only	Previous Infections or Coinfections		
				Bacterial	Viral	Fungal
Perry et al., 1995 [51]	6	+	+	−	−	−
		+	+	−	−	−
		+	+	−	−	−
		+	+	−	−	−
		−	+	−	−	−
		−	+	−	−	−
Sharma et al., 2000 [52]	34	34−	25+	9+	34−	34−
Roters et al., 2001 [53]	1	+	−	+	−	−
Tabin et al., 2001 [29]	2	−	+	−	−	−
		−	−	−	*Herpes* *simplex*	−
Georgakopoulos et al., 2006 [54]	1	+	+	−	−	−
Stemberger et al., 2007 [55]	1	+	+	−	−	−
Elabjer et al., 2009 [56]	1	+	−	+	−	−
Shukla et al., 2012 [30]	5	+	−	−	*Herpes* *simplex*	−
		+	+	−	−	−
		+	−	+	−	−
		+	−	−	*Herpes* *simplex*	−
		+	+	−	−	−
Kwok et al., 2017 [46]	1	+	+	−	−	−
Sun et al., 2020 [57]	1	+	−	+	*Herpes* *simplex*	−
Lin et al., 2023 [58]	1	+	+	−	−	−
	54	16	38	13	4	0

Differences in the immune response and virulence might have an impact on the variable clinical presentation. In their work, Kurbanyan et al. showed that corneal innervation density, nerve length, and branching are significantly lower in cases of active fungal infection or acanthamoebiasis [27]. Moreover, it is suggested that the loss of corneal nerves in AK and fungal keratitis (FK) seems to be greater compared to the changes observed in herpetic keratitis. However, the cause of these changes must be investigated even more thoroughly to confirm the involvement of pathogens in their formation. As a result of eye infection with the *Herpes simplex* type 1 virus, corneal hypoesthesia is observed, while patients with AK often experience severe pain. Such symptoms can be explained by abnormalities and hypersensitivity in the corneal nerves’ survival or regeneration. This pain may also be triggered by cytokines (such as interleukin-1) or nociceptors [59,60].

Five months before the visit to our hospital, the patient was diagnosed with *Herpes* keratitis, which was treated. This prior treatment may have contributed to a reduction in corneal sensitivity. These circumstances can explain the painless course of the disease that caused a considerable delay in the proper diagnosis and treatment in our patient. In addition, it led to the avoidable loss of visual acuity before transplantation and, finally, the transplant failure. The corneal graft was performed in our patient due to the lack of response to the treatment and worsening of the local condition.

Perry et al. draws attention to the symptom of reduced corneal sensation in patients with keratitis caused by *Acanthamoeba*, which very often results in an incorrect diagnosis, usually herpetic keratitis, a delay in treatment and increased morbidity. In the described cases (six patients), a marked decrease in corneal sensation values was observed in all patients using a Cachet–Bonnet esthesiometer (Luneau, Paris, France) [51].

Until now, there have been no standard guidelines for treating acanthamoebiasis or treatment strategies discussed in case reports. According to the literature, to date, several randomized, controlled clinical trials have been conducted on the treatment of AK. There were no differences in the effectiveness of monotherapy with polyhexamethylene biguanide and chlorhexidine. However, combined therapy with biguanides, diamidine derivatives and antibiotic ointments is recommended [59,60]. Moreover, therapeutic penetrating keratoplasty might be considered when the infection escalates to the paracentral corneal stroma, despite maximum antiamoebic therapy [61]. The rate of corneal transplantation performed to control *Acanthamoeba* keratitis ranges from 5% to 68% [62]. The surgical treatment should be delayed until the eye is not inflamed and after completion of the antiacanthamoebic treatment due to the poor prognosis and the high risk of graft failure [63]. The post-operative complications after keratoplasty, in addition to rejection of the graft, include recurrence of the *Acanthamoeba* infection, other infections, glaucoma, cataract, wound leak and irregular astigmatism. Roozbahani et al. observed 75% of graft failures, 50% of cataract, 17% of uncontrolled glaucoma, and 8% of *Acanthamoeba* reactivation in a study group of therapeutic penetrating keratoplasty for *Acanthamoeba* keratitis [64]. Unfortunately, graft failure and cataract development were observed in our patient. The next stage of therapy, a combined surgery including corneal transplantation with cataract phacoemulsification and intraocular lens implantation, was carried out without complications. It was found that diagnosis made within 18 days of symptom onset and initiation of antiamoebic therapy resulted in a better final BCVA after the completion of treatment and eliminated the need for urgent and elective penetrating keratoplasty [65].

It has been suggested that in vivo corneal confocal microscopy (IVCM), which allows a detailed examination of the corneal subbasal nerve plexus and the determination of the degree of corneal damage resulting from infectious keratitis, may not be sensitive enough to detect deep corneal lesions and confirm *Acanthamoeba* infection when diagnosing keratitis [24,57]. In contrast, scleral scattering slit-lamp microscopy, an advanced diagnostic modality that enhances the detection of early and subtle keratitic changes, may offer complementary diagnostic information that facilitates the identification of features characteristic of AK.

Kent et al., describing a painless case of AK, suggest that the reason for the absence of a pain symptom is related to the severity of the accompanying scleritis [30]. In the case of suspected *Acanthamoeba* infection, including mixed infections, taking into account typical symptoms and causes, but also the atypical painless course of the disease, Sun et al. recommended considering and, if necessary, implementing appropriate treatment, even before confirmation by laboratory test results [57].

## 4. Conclusions

In conclusion, *Acanthamoeba* keratitis should be included in the differential diagnosis of keratitis, even in the absence of the classical symptom of severe ocular pain, especially in contact lens wearers and patients who have had herpetic keratitis. Data from the literature and our observations confirm that cornea infection with the *H. simplex* type 1 virus causes nerve degeneration, which translates into a painless course of *Acanthamoeba castellanii* infection. This may be the reason for an incorrect diagnosis and treatment that may further change the clinical picture of the disease and delay the proper therapy [30,48,49,50]. As a consequence, the patient’s quality of life is significantly reduced due to prolonged poor visual acuity and illness, which generates additional costs in terms of medical care.

## Figures and Tables

**Table 1:** Results of the genotyping of *Acanthamoeba* sp. from water and clinical samples.

Sampling	Isolate, Accession No.	Published Sequences in GeneBank			
		Accession No.	Sampling, Isolate	Region of Origin	References
Corneal scraping	Ac55, KP120880	GQ889265	Liver of a Temminck’s tragopan, *Acanthamoeba* sp., genotype: T4 CDCV600	USA	Visvesvara et al., 2010 [36]
		KF318460	Corneal surface tissue, *Acanthamoeba* sp., 1 FRC-2013	Brazil	Mafra et al., 2013 [37]
		EU377583	Biofilm, *Acanthamoeba* sp., CRIB53	Switzerland	Corsaro et al., 2009 [38]
		DQ087296	Contact lenses and contact lens case, *Acanthamoeba* sp., S6	France	Yera et al., 2008 [39]
		DQ087297	Corneal scraping, *Acanthamoeba* sp., 222BAL	France	Yera et al., 2007 [40]

## Data Availability

The raw data supporting the conclusions of this article will be made available by the authors on request.

## Supplementary Materials

The following supporting information can be downloaded at https://www.mdpi.com/article/10.3390/jcm14134763/s1.

## Abbreviations

The following abbreviations are used in this manuscript:

AK	*Acanthamoeba* keratitis
BCVA	Best corrected visual acuity
FK	Fungal keratitis
HSK	*Herpes simplex* keratitis
IOP	Intraocular pressure
IVCM	In vivo corneal confocal microscopy
NK	Neurotrophic keratopathy
