# Supplementary material for: Complicated Diagnosis and Treatment of Rare Painless Acanthamoeba Keratitis
Source: J Clin Med. 2025 Jul 5;14(13):4763. doi: 10.3390/jcm14134763 (PMC12251361; doi:10.3390/jcm14134763)
Supplement: Supplementary file 1 [file jcm-14-04763-s001.zip › jcm-3557653-supplementary.pdf]

SUPPLEMENTARY MATERIAL

Complicated diagnosis and treatment of rare painless *Acanthamoeba* keratitis

Dominika Wróbel-Dudzińska [1], Marta Ziaja-Sołtys [2,*], Beata Rymgayłło-Jankowska [1], Monika Derda [3], Robert Klepacz [4], Daniel Zalewski [2], Tomasz Żarnowski [1] and Anna Bogucka Kocka [2]

[1] Department of Diagnostic and Microsurgery of Glaucoma, Medical University of Lublin, Chmielna 1 Street, 20-079 Lublin, Poland; dominikawrobeldudzinska@umlub.pl (DW-D); beata.rymgayllo-jankowska@umlub.pl (BR-J); tomasz.zarnowski@umlub.pl (TŻ)
[2] Department of Biology with Genetics, Medical University of Lublin, Witolda Chodźki 4A Street, 20-093 Lublin, Poland; marta.ziaja-soltys@umlub.pl (MZ-S); anna.bogucka-kocka@umlub.pl (AB-K); daniel.zalewski@umlub.pl (DZ)
[3] Department of Biology and Medical Parasitology, Poznań University of Medical Sciences, H. Swiecicki Street 4, 60-781 Poznań, Poland; mderda@ump.edu.pl (MD)
[4] Department of Clinical Pathology, Medical University of Lublin, Jaczewskiego 8B Street, 20-090 Lublin, Poland; robert.klepacz@umlub.pl (RK)
* Correspondence: marta.ziaja-soltys@umlub.pl; Tel.: +48 81 448 7235

Molecular identification of *Acanthamoeba* sp. using PCR method

DNA amplification was performed using genus-specific primers previously described by Schroeder et al. [1]. A set of primers that included the forward JDPI (5’GGCCCAGATCGTTTACCGTGAA’3) and the reverse primer JDP2 (5’TCTCACAAGCTGCTAGGGAGTCA’3) was used for genetic characterization targeting an approx. 450 bp fragment of the *Acanthamoeba* 18S ribosomal RNA (rRNA) gene. The amplification involved the use of 25 μl of a suspension of the following reagents: 2.5 mM $MgCl_2$, 0.6 - 1 μM of each primer, 0.2 mM of each deoxynucleotide triphosphate, and 0.5 U of AmpliTaq Gold DNA polymerase (QIAamp DNA Mini Kit, QIAGEN, Hilden, Germany). A clinical isolate of *Acanthamoeba castellanii* belonging to the T4 genotype isolated from a keratitis patient (ATCC 50374) was used as a positive control. A negative control consisting of the reaction mixture without a DNA template was included. Polymerase chain reaction (PCR) was carried out using a GeneAmp 2400 thermocycler (Applied Biosystems, Foster City, CA, USA). After a denaturation step at 94°C for 5 min, 35 cycles of amplification were performed as follows: 1 min at 94°C, 1 min at 56°C, and 1 min at 72°C, followed by a final extension of 72°C for 10 min. Two microliters of sterile water were used as a negative control. The PCR products were analyzed on 1% agarose gel (Gibco BRL, UK) stained with ethidium bromide (Merck, Germany). The gel images were illuminated with ultraviolet (UV) light and captured using a gel documentation system (Transilluminator, Foto/Prep, USA).

Molecular identification of *Acanthamoeba* sp. using sequencing of PCR products

The PCR products were sequenced in both directions with the same set of primers. Sequencing was performed with BigDye Terminator v3.1 on an ABI Prism 3130XL Analyzer (Applied Biosystems, Foster City, CA, USA). The trace files were checked and edited using FinchTV 1.3.1 (Geospiza Inc., Seattle, USA).

The contigs were aligned and manually assembled in GeneDoc v. 2.7.000 (https://nrbsc.org/gfx/genedoc/). Sequences were analyzed using the Chromas program (https://technelysium.com.au/wp/chromas/). The gene sequence fragments of the *Acanthamoeba* isolates were then compared with the reference sequences deposited in Gen-Bank (National Center for Biotechnology Information).

1. Schroeder, J.M.; Booton, G.C.; Hay, J.; Niszl, I.A.; Seal, D.V.; Markus, M.B.; Fuerst, P.A.; Byers, T.J. Use of Subgenic 18S Ribosomal DNA PCR and Sequencing for Genus and Genotype Identification of Acanthamoebae from Humans with Keratitis and from Sewage Sludge. *J Clin Microbiol* **2001**, *39*, 1903–1911, doi:10.1128/JCM.39.5.1903-1911.2001.
